# A Case Series of Breast Metastases from Different Extramammary Malignancies and Their Literature Review

**DOI:** 10.1155/2019/9454201

**Published:** 2019-01-08

**Authors:** Liliana Moreno-Astudillo, Yolanda Villaseñor-Navarro, Vyanka Sánchez-Goytia, Fany Porras-Reyes, Alfredo Lara-Mercado, Isabel Sollozo-Dupont

**Affiliations:** ^1^Department of Radiology and Imaging, Instituto Nacional de Cancerología INCan, Ciudad de México, Mexico; ^2^Department of Surgical Pathology, Instituto Nacional de Cancerología INCan, Ciudad de México, Mexico

## Abstract

Metastasis to the breast from all other primary sites is unusual. Twelve patients were diagnosed between 2007 and 2017 at National Cancer Institute, Mexico. Solitary or multiple masses, round or oval, and hypoechoic and solid lesions with posterior acoustic shadowing were patterns commonly reported in these patients; other arrangements include diffuse involvement of the breast simulating an inflammatory carcinoma. The development of a breast metastasis is revealed, in our experience, as a negative prognostic factor. Thus, the radiologist should know about the varied appearance of metastatic breast lesions and provide radiopathological correlations when available.

## 1. Introduction

Prior to the 1990s, most reports regarding metastases to the breast from nonmammary primary tumors were clinical observations or pathological series and provided neither imaging findings nor radiologic information. Fortunately, in more recent decades, imaging of metastases to the breast has been largely investigated, defining useful clues to their diagnosis [1]. For example, there is accumulating evidence that breast metastases manifest most frequently as round or oval masses with circumscribed margins on mammography and as hypoechoic masses with microlobulated or circumscribed margins and posterior acoustic enhancement on ultrasound [2]. However, in light of our experience, metastatic breast lesions show variable imaging features that depend on the origin and location of the primary tumors, and their differentiation from primary tumors, or from a benign condition, is difficult. Misleading radiographic evaluations may yield false-negative results, particularly in healthy patients, or they may result in a diagnostic delay for cancers of unknown primary origin [3]. In this case series, we present and illustrate the mammographic and sonographic appearance of breast lesions from extramammary malignancies, providing morphological clues in accordance with primary cancer when available.

## 2. Clinical Examples

### 2.1. Breast Metastases from Ovarian Carcinoma

Intra-abdominal spread manifesting as peritoneal carcinomatosis represents the typical course of ovarian metastasis, whereas distant lesions are seen most commonly in the lung, liver, or pleura and rarely involve the breast. As a result, Medline features fewer than 120 reports of breast metastases of ovarian origin since the first case described by Sitzenfrey [4]. Of predictive interest, at least 70% of patients with breast metastases arising from ovarian carcinoma have papillary serous carcinoma [5]. Nevertheless, clear-cell carcinoma, granulosa cell tumors, and dysgerminoma are other histological types that affect the breast [6]. Further, numerous datasets have shown that the age at diagnosis may vary widely, ranging from 30 to 80 years [7–10].

It is noteworthy to mention that coexisting breast and ovarian malignancies generally occur in carriers of the* BRCA* mutation [11]. Thus, the diagnosis of a breast tumor in patients with ovarian carcinoma might be an indication to evaluate* BRCA* status [12, 13]. In addition, a loss of p53 function is seen more often in* BRCA1*-associated tumors than in sporadic breast or ovarian tumors, which implies blockage of genomic damage repair, which can have subsequent negative impacts on overall survival and disease-free survival [14, 15].

**Figure 1:**
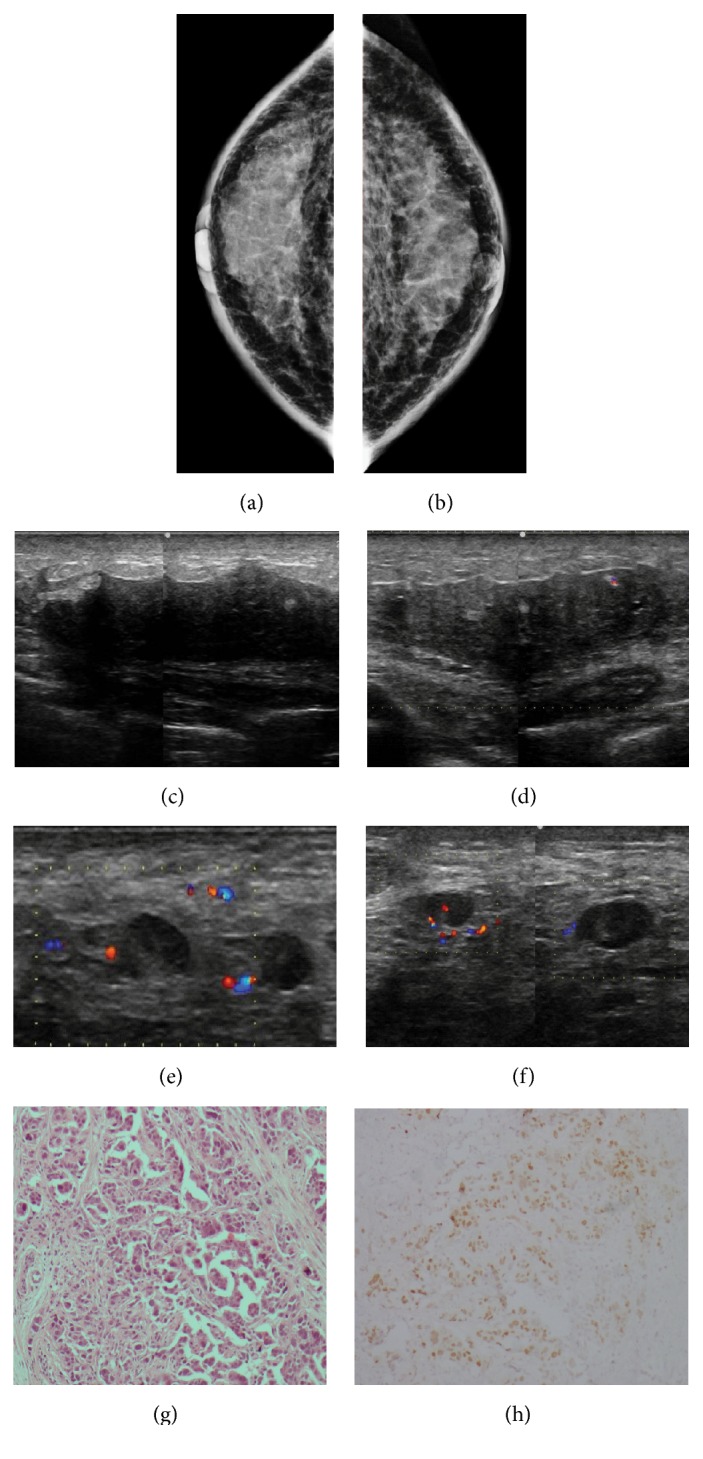
**Case** **1**. A 66-year-old-woman diagnosed with primary ovarian carcinoma with signet ring cells. On initial presentation, the patient showed breast lymphedema. Mammogram revealed abnormal skin enhancement, thickening, and edema consistent with inflammatory changes (**a–b**). Breast sonography exposed architectural distortions with a posterior combined pattern (**c–d**). Bilateral axillary lymph nodes with irregular cortical thickening were also found (**e–f**). Metastatic signet ring cell carcinoma to the breast was confirmed following a histopathological analysis of the breast and lymph node specimens. HE staining and immunohistochemistry analysis of the metastases. HE staining revealed the malignant cells in the breast tumor (**g**), and the immunohistochemistry analysis indicated that the cells were positive for PAX8 (**h**).

**Figure 2:**
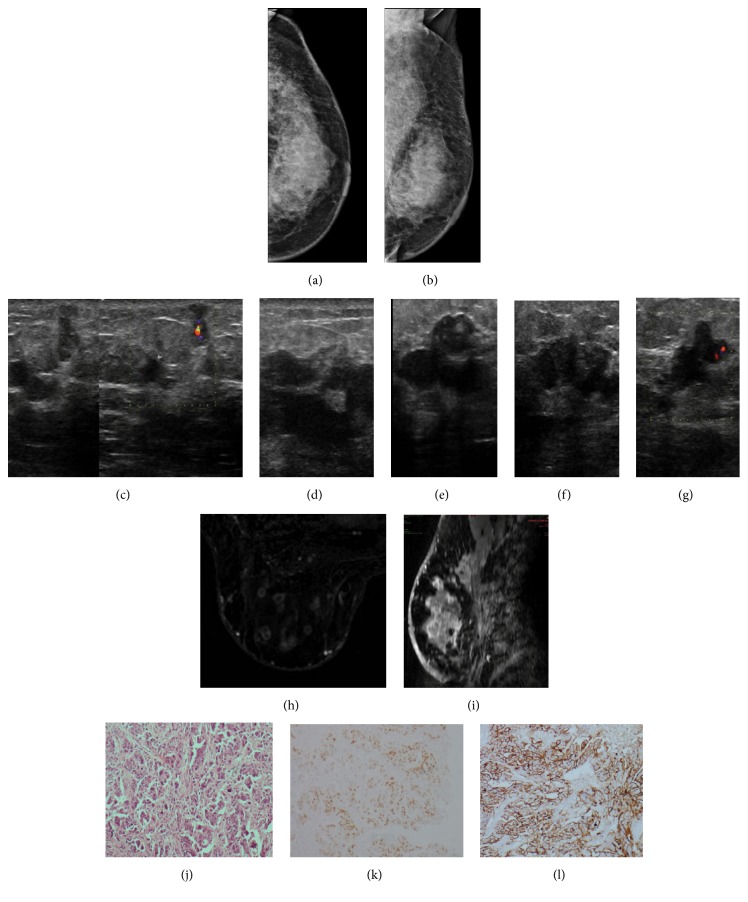
**Case** **2**. A 60-year-old woman diagnosed with papillary serous ovarian carcinoma. A palpable mass in the left breast was noticed by the patient 2 years after initial diagnosis. Mammogram revealed dermal thickening and diffusely increased breast density (**a–b**). A distortion was evident in the breast tail as well, indicating skin retraction (**b**). Grayscale and Doppler ultrasound showed bilateral hypoechoic irregular masses with angular margins. A nonparallel orientation and central vascularity were observed in some masses. Multifocal and diffuse distribution of the lesions was noted on ultrasound (**c–g**). Similarly, subtracted images from magnetic resonance demonstrated multiple irregular masses with circumscribed margins and rim enhancement (**h**). Conglomerate lesions infiltrating the major pectoralis muscle were also exposed (**i**). HE staining and immunohistochemistry analysis of the metastases. HE staining revealed the malignant cells in the breast tumor (**j**), and the immunohistochemistry analysis indicated that the cells were positive for PAX8 (**k**) and CA125 (**l**).

The primary clinical signs of patients with metastatic ovarian breast cancer include solitary or multiple palpable lesions that grow rapidly. Also, ovarian cancer affecting the breast may be found to be inflammatory carcinoma with erythema and thickening of the skin (**Case** **1** **, Figures 1(a)–1(d); Case** **2** **, Figures 2(a) and 2(b)**), and/or with the peau d'orange sign [7–10]. Axillary lymphadenopathy is another feature observed in these patients (**Case** **1** **, Figures 1(e)–1(f); Case** **4** **, Figure 4(d)**) [8, 9], which may be related to the drainage of ovarian cancer from the intra-abdominal to axillary lymph nodes, and then to the lymphatic channels in the breast [9].

**Figure 3:**
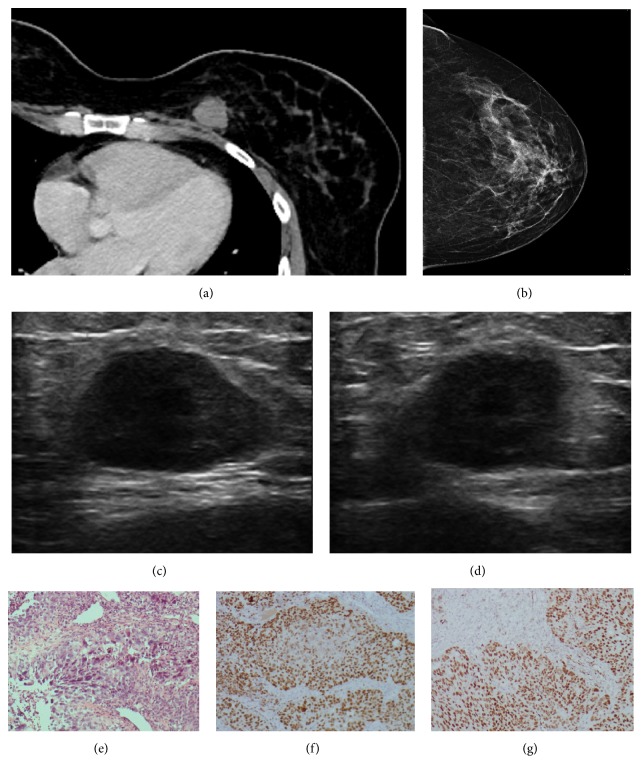
**Case** **3**. A 41-year-old woman diagnosed with a low-differentiated carcinoma. Contrast-enhanced computed tomography (CT) scan demonstrated an incidental finding consistent with a mass on the right breast (**a**). Although this lesion was palpable, it was not detectable on mammography (**b**). Then, an ultrasound was performed, showing an oval mass with parallel orientation, indistinct margins, a heterogeneous echo pattern, and posterior acoustic enhancement (**c–d**). HE staining and immunohistochemistry analysis of the metastases. HE staining revealed the malignant cells in the breast tumor (**e**), and the immunohistochemistry analysis indicated that the cells were positive for PAX8 (**f**) and WT4 (**g**).

**Figure 4:**
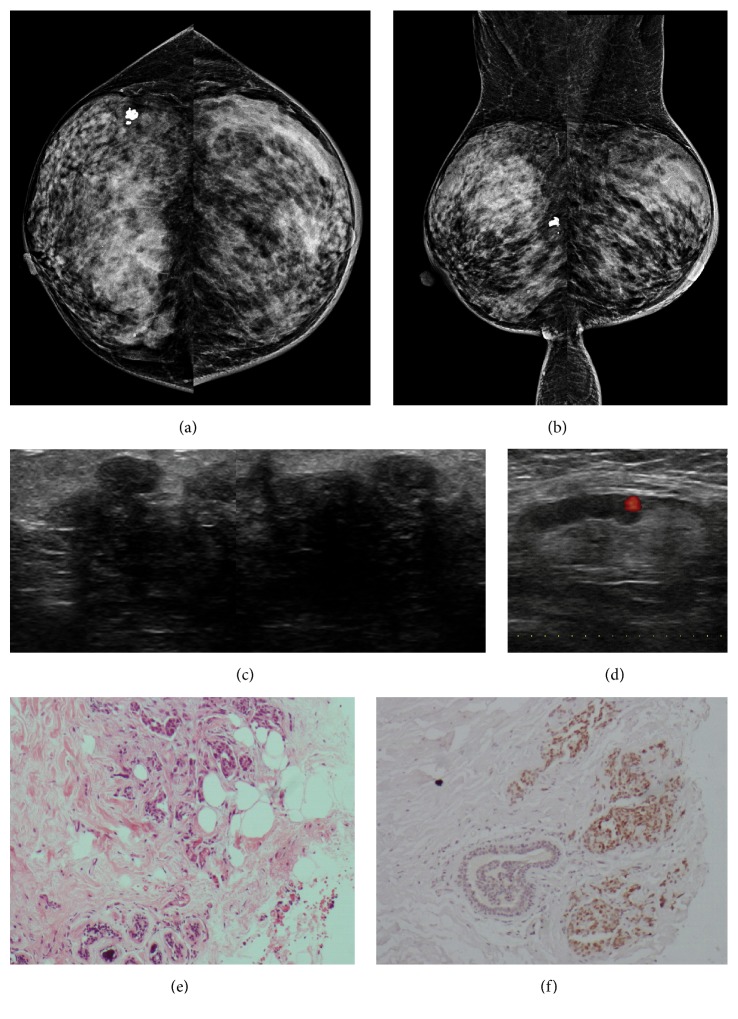
**Case** **4**. A 56-year-old woman diagnosed with ovarian adenocarcinoma. One month after diagnosis, the patient presented with palpable axillary adenopathy. Mammogram showed dermal and trabecular thickening in the left breast, with diffusely increased density of the mammary tissue (**a–b**). On ultrasound, an irregular hypoechoic mass with indistinct margins was revealed (**c**). An axillary lymph node with cortical indentation was also observed by ultrasound (**d**). HE staining and immunohistochemistry analysis of the metastases. HE staining revealed the malignant cells in the breast tumor (**e**), and the immunohistochemistry analysis indicated that the cells were positive for PAX8 (**f**).

Furthermore, imaging studies show that nodular metastases usually appear as large, intramammary masses with microlobulated margins on mammography [7, 16, 17]. On ultrasound, ovarian metastases typically appear as oval masses with an indistinct, noncircumscribed margin (**Case** **3** **, Figures 3(a)–3(d)**) and posterior acoustic shadowing (**Case** **4** **, Figures 4(a)–4(c); Case** **2** **, Figures 2(c)–2(g)**) [16]. As stated by Abbas et al. and Tempfer et al., architectural distortion and microcalcifications in these lesions might also be evident during radiological evaluations (**Case** **2** **, Figures 2(b) and 2(i)**) [6, 16]. When microcalcifications are presented, a serous psammomacarcinoma of the breast is suspected [16].

In summary, the differential diagnosis of ovarian metastatic breast cancer includes recognizable but unspecific imaging patterns [18]. In any case, making a definitive pathological diagnosis is essential, as primary breast cancer and extramammary metastases arising from a malignant tumor other than breast cancer require different therapies [16]. Fortunately, metastatic lesions of the breast that arise from ovarian carcinoma are rare, with an overall incidence of 0.07% [8]. Nevertheless, >90% of affected patients succumb to the disease with survival times ranging from 1 to 52 months [5, 8, 9].

### 2.2. Breast Metastases from Melanoma

**Figure 5:**
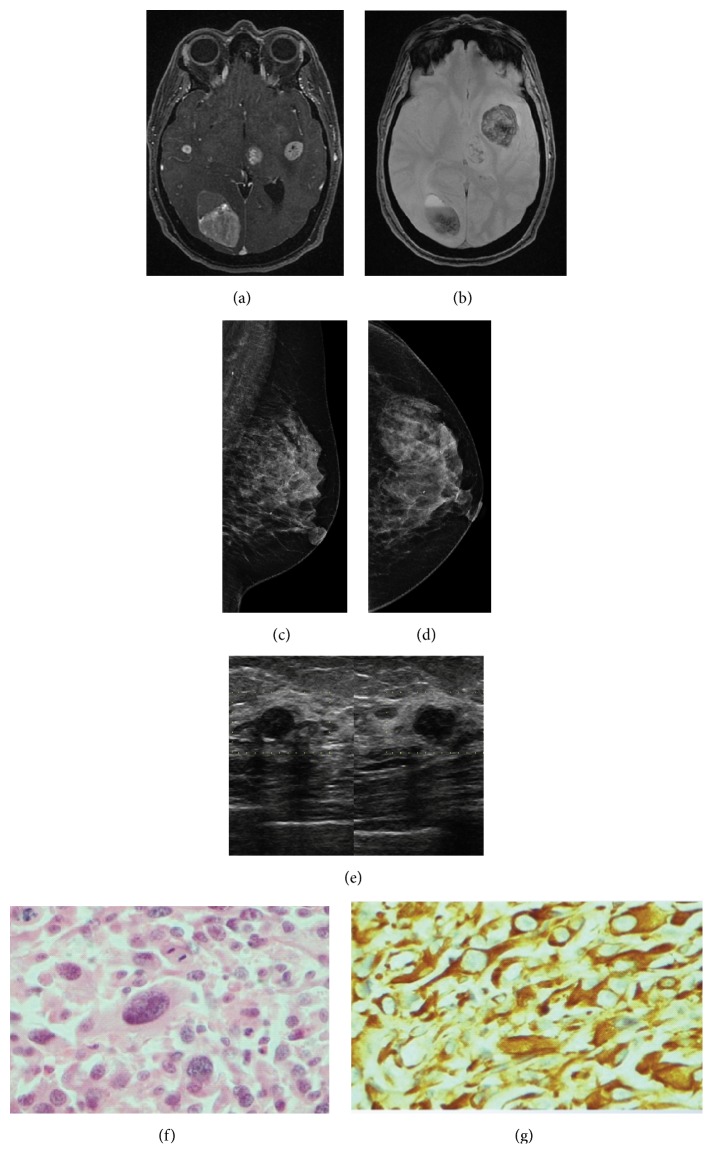
**Case** **5**. A 45-year-old woman with metastatic melanoma that presented as an isolated breast tumor. Upon interrogation, the patient reported a 1-month history of migraine and attacks of vertigo. Magnetic resonance imaging showed multiple heterogeneous enhancing masses suggesting the presence of metastatic disease (**a**). Intracranial hemorrhage focus was corroborated by fluid–fluid levels on gradient*-*echo imaging (**b**). Following the suspicion of metastatic disease, mammography was performed, demonstrating a subtle increase in density in both breasts (**c–d**). On ultrasound, an oval, hypoechoic mass with multilobulated margins was shown in the left breast (**e**). HE staining and immunohistochemistry analysis of the metastases. HE staining revealed the malignant cells in the breast tumor (**f**), and the immunohistochemistry analysis indicated that the cells were positive for melan-A (**g**).

The incidence of cutaneous melanoma has been consistently rising by 3%–7% annually, increasing the number of skin cancer-related deaths in Caucasian populations [19]. The data suggest that ~20% of affected patients will develop metastatic disease in the liver, lung, and brain (**Case** **5** **, Figures 5(a) and 5(b)**) [20]. Conversely, cases of metastases to the breast that arise from melanoma are rare, constituting approximately 1.3%–2.7% of all malignant mammary tumors. Despite their low prevalence rates, these metastases must be considered in any patient with a breast lump and any history of a known primary malignant tumor [21].

According to Ravdel et al., the age at diagnosis for patients with metastatic melanoma to the breast ranges from 27 to 70 years, with a median age at diagnosis of 41.4 years [22].

The clinical data showcase that melanoma in the breast is generally asymptomatic, although it may be palpable and present as dense, well-circumscribed nodules. As stated by some authors, this disease may proliferate, arising like inflammatory breast cancer in some instances [23, 24]. With respect to location, metastases that arise from melanomas are generally found in the upper-outer quadrant of the breast and in the superficial subcutaneous tissues (**Case** **7** **, Figures 7(c)–7(f)**), which may be related to the abundant blood supply in the subcutaneous fat and skin when compared with the breast parenchyma [25].

**Figure 6:**
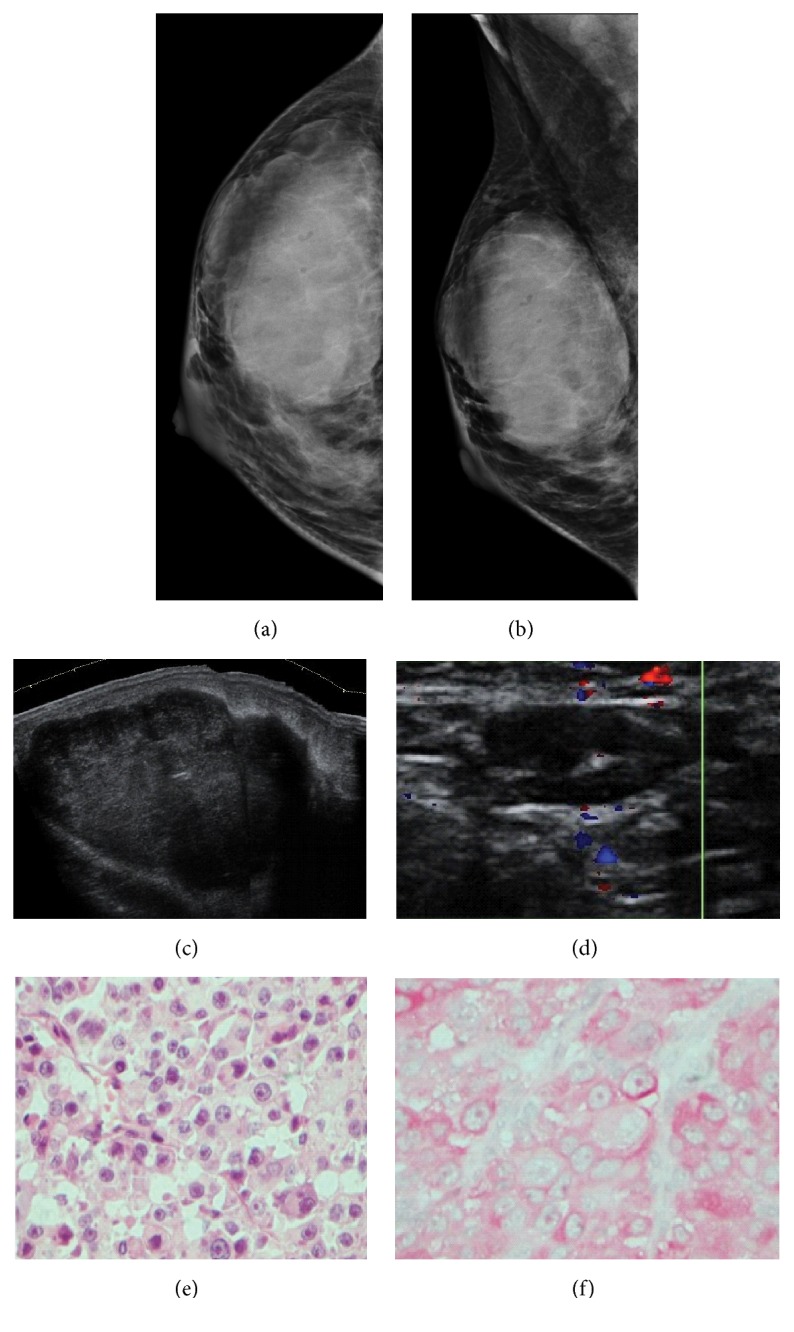
**Case** **6**. A 20-year-old woman with nodular melanoma. The patient presented with a 9-month history of nevus in the neck around the middle line. The woman was lost to follow-up before surgery, and 10 months later, she returned with a palpable mass in the right breast. Mammogram showed an oval mass with circumscribed margins (**a–b**). Ultrasound images demonstrated a sizeable oval mass with a parallel orientation, circumscribed margins, and absent posterior acoustic findings (**c**). A lymph node with irregular cortical enhancement was also exhibited (**d**). HE staining and immunohistochemistry analysis of the metastases. HE staining revealed the malignant cells in the breast tumor (**e**), and the immunohistochemistry analysis indicated that the cells were positive for HMB45 (**f**).

**Figure 7:**
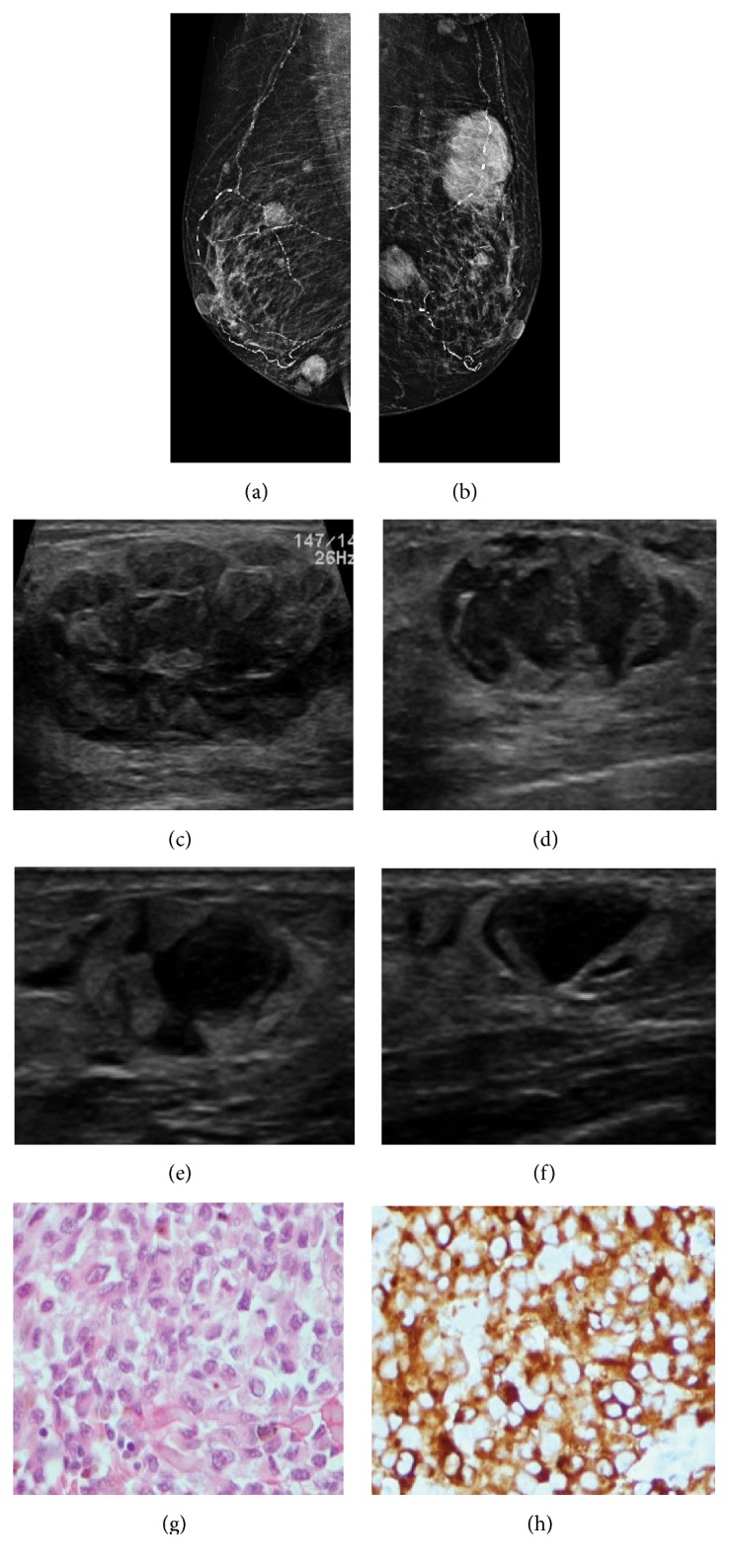
**Case** **7**. A 71-year-old woman with malignant melanoma. Two years after diagnosis, the patient presented with a palpable mass in the left breast. Mammogram showed many oval masses with circumscribed margins (**a–b**). These findings were corroborated on ultrasound, exhibiting oval masses with indistinct and microlobulated margins and a heterogeneous echo pattern (**c–f**). Edema was also evident (**e–f**). Metastatic melanoma to the breast was confirmed following a histopathological analysis of the breast specimen. HE staining and immunohistochemistry analysis of the metastases. HE staining revealed the malignant cells in the breast tumor (**g**), and the immunohistochemistry analysis indicated that the cells were positive for HMB45 (**h**).

Furthermore, studies reporting morphological findings in cases of melanoma that affect the breast are in agreement that mammography will often detect unique or multiple well-defined nodular opacities (**Case** **6** **, Figures 6(a)–6(c)**;** Case** **7** **, Figures 7(a) and 7(b)**) [21, 22, 26, 27]. Accordingly, oval, hypoechoic masses with lobulated or well-circumscribed margins and a well-defined posterior wall are the most common patterns found on ultrasound (**Case** **5** **, Figures 5(c)–5(e)**) [21, 28]. These features differ from those of primary breast malignancies, which are usually irregular with posterior acoustic shadowing; as such, melanoma metastases may be relatively benign [28]. Further, other features of breast carcinomas, including calcifications or architectural distortion, are distinctively absent in cases of melanoma that metastasize to the breast [21].

In conclusion, mammography and ultrasonography findings are not pathognomonic of the metastatic foci that arise from melanoma, requiring a tissue biopsy diagnosis to confirm their presence. In any case, the diagnosis may be straightforward if there is a clinical history of melanoma.

Unfortunately, the 5-year overall survival for patients with metastatic melanoma is about 20%, with the median survival time ranging from 6 to 9 months; the prognosis is worse with a bilateral metastatic process [28].

### 2.3. Breast Metastases Arising from Lymphoma

Breast lymphoma is a very rare entity, accounting for only 0.1%–0.5% of all breast cancer cases [29, 30]. Further, 0.38%–0.7% of all non-Hodgkin lymphomas (NHL) and 1.7%–2.2% of extranodal NHL cases result in breast metastases [29]. The histological type is predominantly B-cell lymphoma, accounting for 85%–95% of all cases, followed by T-cell, Burkitt, mucosa-associated lymphoid tissue lymphoma (MALT), and extranodal natural killer lymphoma nasal types (ENKTL) affecting 5%–15% of patients [30]. In a retrospective study of 204 cases of breast lymphoma, it was found that the age at diagnosis ranges from 50 to 60 years, with a median age at diagnosis of 71 years [31].

Breast lymphoma may occur as either primary or secondary breast involvement [32, 33]. The definition of primary breast lymphoma (PBL) comprises only stage I (lymphoma limited to the breast) and stage II tumors (lymphoma confined to the breast and axillary lymph nodes), whereas in secondary breast lymphoma (SBL), the breast is involved, but through the secondary infiltration of a systematic disease [30, 33]. Distinguishing between PBL and SBL is vital since differences exist in tumor biology and aggressiveness [34]. Nonetheless, both entities typically display similar clinical and radiographic appearances [35].

According to some authors, breast lymphoma commonly occurs as a breast mass given its fast growth [32, 36]. However, the clinical data expose how changes in the subcutaneous tissue or the skin, or ipsilateral lymph node enlargement, may be present (**Case** **9** **, Figures 9(a), 9(b), 9(i), and 9(j)**) [30, 35]. A further observational point in the literature is the right breast affectation in both PBL and SBL [30]. However, several reports indicate that the left breast may be equally as affected as the right [35, 37]. For example, in a total of 36 lymphoma cases, Surov et al. reported that left and right breast involvement accounted for 39% and 33% of cases, respectively, with no significant differences in bilateral involvement, which affected 28% of patients [35].

**Figure 8:**
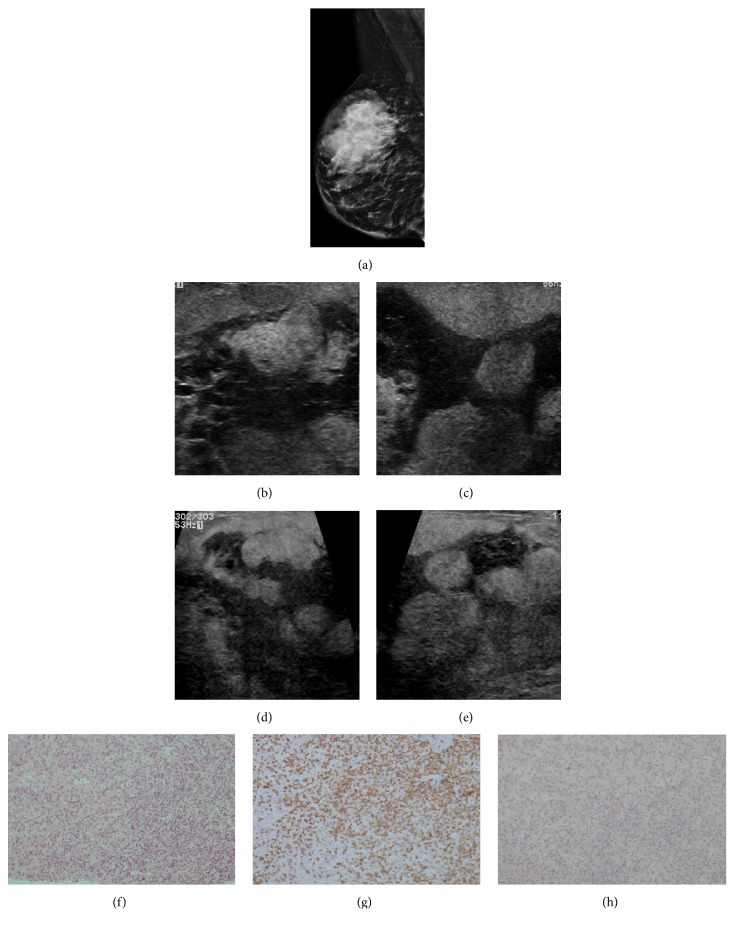
**Case** **8**. A 57-year-old-woman with Epstein–Barr virus (EBV)-associated, extranodal natural killer (NK)-cell lymphoma of nasal type. In 2006, the patient was diagnosed with breast cancer, which was treated with sentinel lymph node (SLN) biopsy and a total mastectomy. In 2016, the patient was admitted to our institution for an unusual nasal blockage and rhinorrhea over a 1-month period. Upon interrogation, the patient also reported progressive loss of vision in the left eye, which was associated with a mass. Notably, a palpable mass was appreciated in the right breast at the time of the evaluation (**a**). Mammogram showed an irregular mass with indistinct margins occupying the upper-outer quadrant of the right breast (**b–e**). Grayscale ultrasound demonstrated an irregular, solid mass with indistinct and angular margins, a heterogeneous echo pattern, and posterior features with a combined pattern. HE staining and immunohistochemistry analysis of the metastases. HE staining revealed the malignant cells in the breast tumor (**f**), and the immunohistochemistry analysis indicated that the cells were positive for CD45 (**g**), and negative for CKAE1-AE3 (**h**).

**Figure 9:**
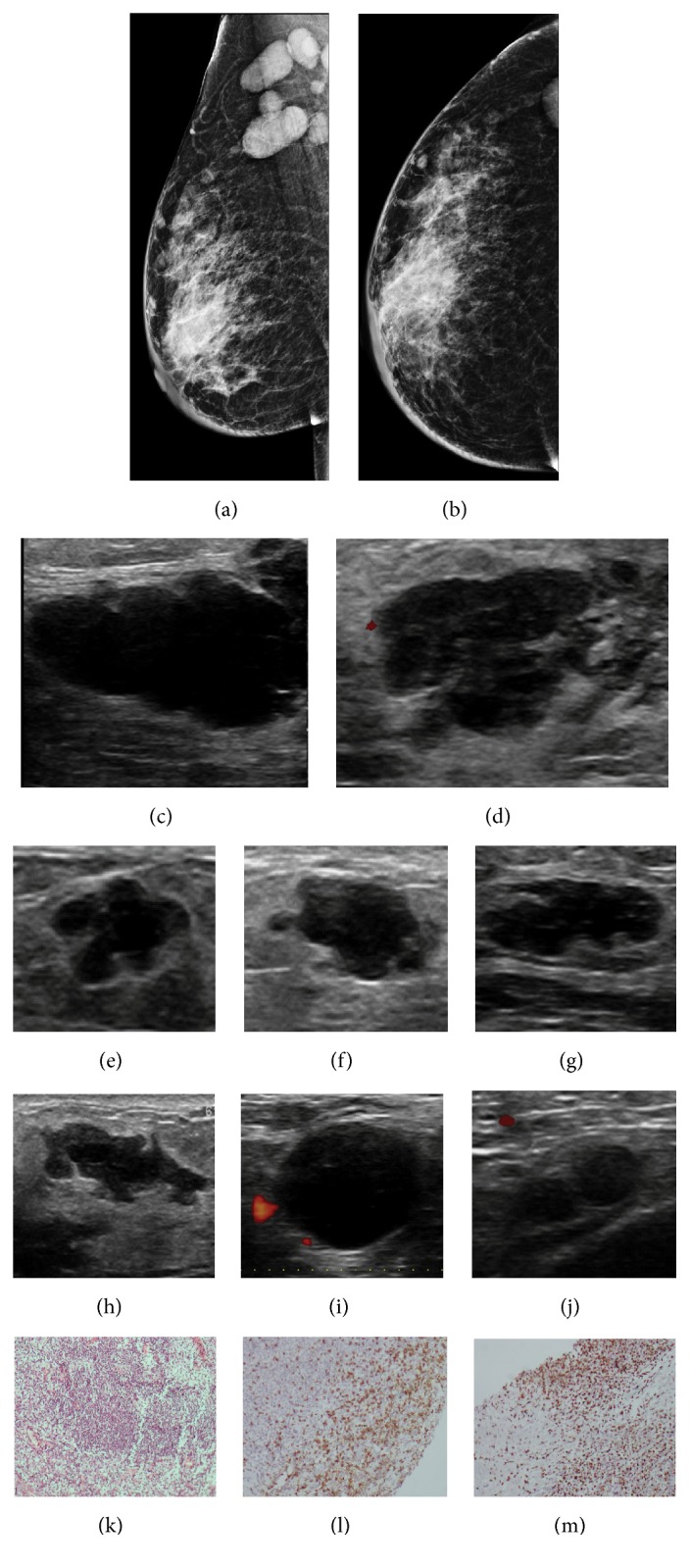
**Case** **9**. A 62-year-old woman with large B-cell lymphoma of germinal center origin. The patient was admitted to our institution for 3 months given the presence of a palpable mass in the right breast (**a–b**). On mammography, multiple focal asymmetries in the retroareolar region were noted. Additionally, mammograms showed skin thickening and oval masses located on the upper-outer quadrant that were isodense and featured circumscribed margins (**c–h**). Multiple irregular masses with multilobulated margins and heterogeneous echo patterns were observed on ultrasound (**i–j**). Both axillary and infraclavicular adenopathies were noticed. HE staining and immunohistochemistry analysis of the metastases. HE staining revealed the malignant cells in the breast tumor (**k**), and the immunohistochemistry analysis indicated that the cells were positive for CD20 (**l**). Additionally, it is shown that Ki-67 protein was expressed in 70% of the tumor cells (**m**).

Imaging studies largely demonstrate that lymphoma affecting the breast is mainly observed as a nodular disease on mammography. According to Yang et al., most of these masses are irregular or oval in shape with indistinct margins (**Case** **8** **, Figures 8(a)–8(e)**) [38]. Nevertheless, oval-shaped nodules with well-circumscribed margins may also occur [39]. Ultrasound studies support the notion that nodular breast lymphoma typically presents as well-defined, oval masses with variable echogenicity, which may be associated with posterior acoustic shadowing and an echogenic rim or onion peel-like rim surrounding lesions; these findings may represent cases of lymphedema (**Case** **9** **, Figures 9(c)–9(h)**) [39]. Other less-common findings include architectural distortions and increased breast density, representing a diffuse infiltration process [30, 32, 35].

Of note, breast lymphoma can go unnoticed on mammography, even in cases of diffuse breast infiltration or the presence of a bilateral dam; breast lymphoma should be considered in all patients being investigated or treated for cases of lymphoma or PBL, as this may alert the radiologist of a possible misdiagnosis.

Another remarkable feature is the poor prognosis of patients with metastatic breast lymphoma; this cancer has a median overall survival of only 29 months [31].

### 2.4. Breast Metastases from the Gastrointestinal Tract

Gastric carcinomas that metastasize to the breast are sporadic. To our knowledge, there are fewer than 50 cases reported in the English literature. Premenopausal women are most commonly affected by the disease, and a previously reported average age at presentation is 47 years [40]. Examination of cases of metastatic gastric carcinoma to the breast reveals an increased percentage of patients affected by colorectal cancer, whereas the most common histological type is the signet-ring cell carcinoma, followed by mucinous carcinoma [41, 42]. Interestingly, signet-ring cell carcinoma is a unique subtype of mucin-producing adenocarcinomas, which can arise from the stomach, colon, and breast; thus, breast specimens obtained from gastric cancer patients should be carefully analyzed during pathology to differentiate between metastasis and a potential primary breast cancer [41, 43].

A further key point in the literature is that a milieu rich in estrogen and estrogen receptors promotes tumorigenesis and the formation of metastatic lesions in gastric cancer patients. As a result, the mammary gland is susceptible to the malignant properties of this cancer [44]. Moreover, germline mutations in the* CDH1* gene that cause hereditary diffuse gastric cancer syndrome (HDGC) are also found in 0.7% of women with breast cancer, suggesting that there is an inherited correlation between diffuse gastric and breast cancers, mainly those of the lobular type [45].

**Figure 10:**
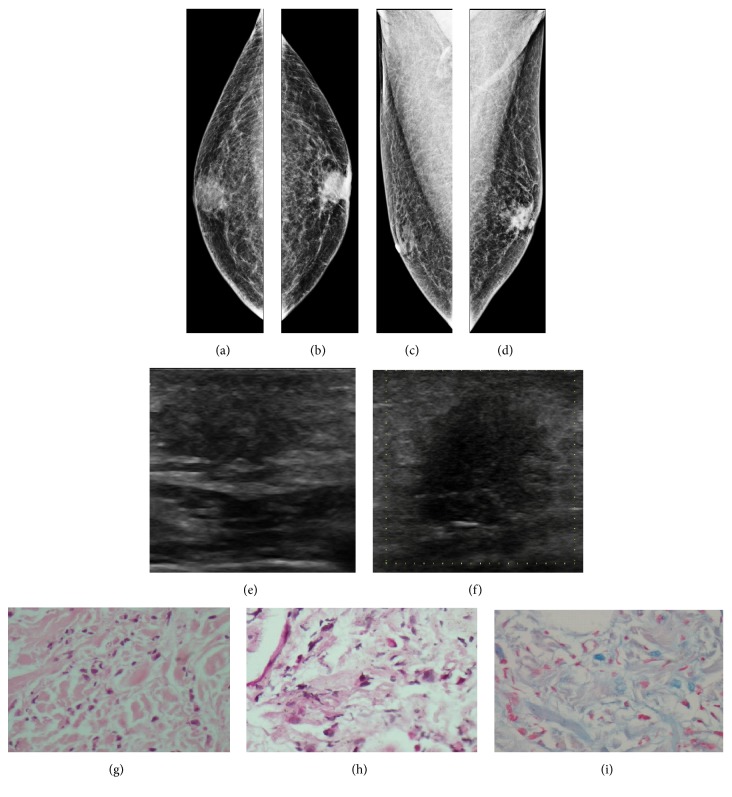
**Case** **1** **0**. A 63-year-old man with gastric signet ring cell carcinoma. One month after diagnosis, the patient presented with a palpable mass in the left breast (**b and d**). Mammogram showed focal asymmetry in the retroareolar region in the left breast and a high-density mass that resulted in nipple retraction (**a–d**). On ultrasound, this corresponded to a suspicious, solid heterogeneous mass with indistinct margins and posterior acoustic shadowing (**e–f**). A comparable lesion was demonstrated in the right breast. HE staining and immunohistochemistry analysis of the metastases. HE staining revealed the malignant cells in the breast tumor (**g**), and the immunohistochemistry analysis indicated that the cells were positive for PAS (**h**) and Alcian blue (**i**).

According to some authors, gastric metastases to the breast usually present as a painless, firm, single mass in the upper-outer quadrant of the breast on clinical examination. However, this disease may also present as multiple nodules or it may exhibit diffuse involvement and feature corresponding skin changes, such as skin thickening or increased consistency [7]. Another remarkable feature is that ~25% of patients with breast metastases have bilateral breast tumors (**Case** **1** **0** **, Figures 10(a)–10(f)**), while axillary lymph node metastases are only reported in ~5% of cases [40].

**Figure 11:**
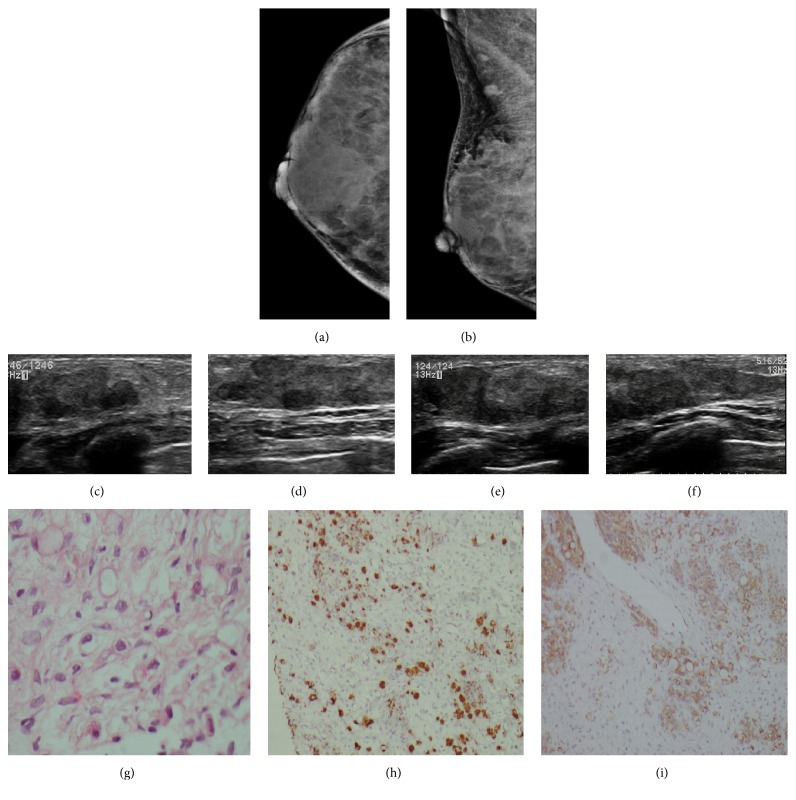
**Case** **11**. A 40-year-old woman with gastric signet ring-cell carcinoma. A solid mass in the upper-outer quadrant of the right breast was detected on initial exploration. (**a–b**) Mammogram showed extremely dense parenchyma of the right breast, which corresponded to multiple pseudonodular, hypoechoic, irregular masses with indistinct margins on ultrasound (**c–f**). HE staining and immunohistochemistry analysis of the metastases. HE staining revealed the malignant cells in the breast tumor (**g**), and the immunohistochemistry analysis indicated that the cells were positive for MUC5AC (**h**) and CK20 (**j**).

Imaging studies show that when the metastatic focus is a mass, mammography findings generally show a round lesion with well-defined margins. These masses can present as benign lesions; however, ill-defined margins may also be evident (**Case** **11** **, Figures 11(a)–11(f)**) [41, 44]. Conversely, only a few cases of microcalcifications associated with breast metastases that arise from gastric cancer are reported in the literature [46]. On sonography, gastric lesions are hypoechoic masses with an irregular shape and indistinct margins (**Case** **1** **0** **, Figures 10(e) and 10(f)**). Increased vascular flow on Doppler may be evident [44].

Mammography and ultrasound can be used to define a relatively small number of findings that may be useful as markers of metastatic breast disease that arise from gastric cancer. However, capturing and understanding the heterogeneity of these lesions may be of paramount importance for studies addressing survival and treatment in metastatic processes. Unfortunately, with the metastatic spread of gastric cancer to the breast, the overall survival is significantly reduced to 1 month, up to a maximum of 18 months [41].

### 2.5. Breast Metastases from the Head and Neck

It has been reported that head and neck carcinomas primarily involve locoregional growth, while the prevalence of distant metastases is around 15%–20% [47]. Advanced-stage primary tumors in the hypopharynx, oropharynx, and oral cavity are associated with the highest incidence of distant metastases [48]. A retrospective study on 832 patients with squamous cell carcinoma of the head and neck exposed that the most frequent metastatic sites are the lungs, followed by the mediastinal nodes, liver, and bones. Breast metastases from the head and neck are rarely reported, with less than 15 cases documented in the English literature to our knowledge. Those cases exhibit an average age at presentation of 47 years (range: 28–73 years), with an overall survival of approximately 10 months [49].

**Figure 12:**
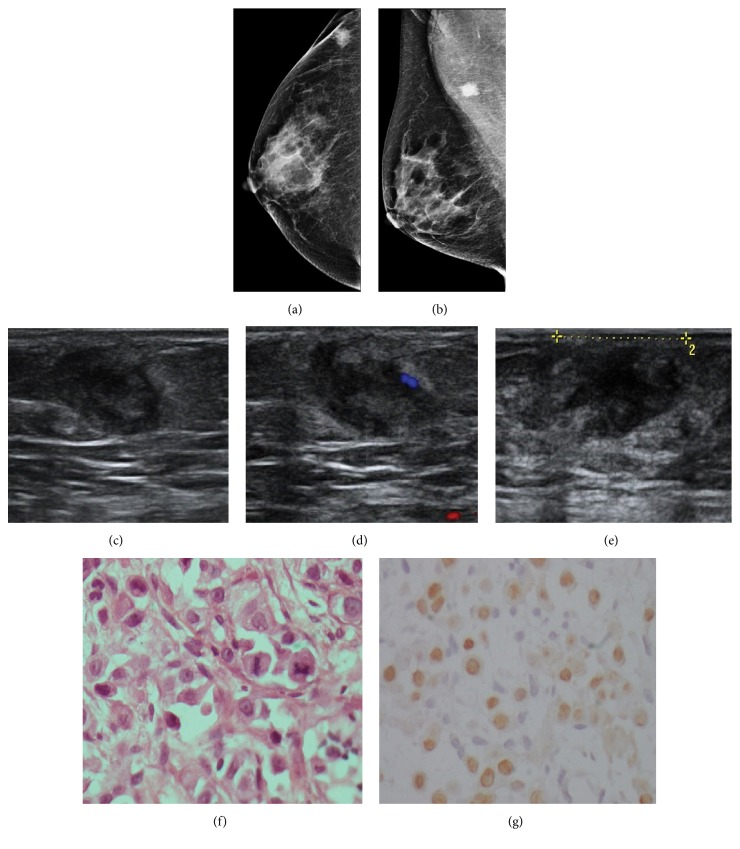
**Case** **1** **2**. 42-year-old woman with carcinoma of the tongue. One year after diagnosis, the patient presented with a palpable mass in the right breast. On mammography, an irregular, isodense mass was noted in the breast tail (**a–b**). An oval mass with circumscribed margins and a heterogeneous echo pattern was seen on ultrasound. The lesion also showed posterior acoustic enhancement (**c–e**), while color Doppler images demonstrated peripheral vascularity (**d**). HE staining and immunohistochemistry analysis of the metastases. HE staining revealed the malignant cells in the breast tumor (**f**), and the immunohistochemistry analysis indicated that the cells were positive for P63 (**g**).

The first clinically detected cases, which were both of the oral cavity, were described by Toombs and Kalisher in 1977; they reported that breast metastasis arising from head and neck cancers appeared like solitary discrete lesions in the breast [50, 51]. However, a remarkable feature found in the literature is that breast metastases from the head and neck were incidentally found in most of the reported cases. Under such circumstances, their clinical presentation is not well characterized. Radiographic descriptions of breast metastases arising from the head and neck are also limited. The case reported by Ascani et al. is one of a few to describe ill-defined, oval masses on mammography in a patient with thyroid follicular carcinoma [50]. Also, Khazai et al. recently reported a case of metastatic salivary duct carcinoma where focal asymmetry in the retroareolar region was the main finding. On ultrasound, these metastases presented as irregular, hypoechoic breast masses that increased suspicion of malignancy (**Case** **1** **2** **, Figures 12(a)–12(e)**) [52].

Finally, it is known that human papillomavirus (HPV) cancers of the oropharynx are associated with more diffuse metastases and spread to uncommon sites such as the breast; this is in contrast with what is found for cancers not related to HPV. Nonetheless, the importance of this and other prognostic factors, such as extracapsular spread, has not been sufficiently recognized in breast cancer-specific survival. Thus, in any patient with a history of head and neck cancer, including that involving HPV, a new breast mass or the presence of a nonspecific finding must always prompt the clinician to investigate the possibility of unusual hematogenous metastases when making a differential diagnosis [51].

## 3. Discussion

It is well established that the metastatic disease to the breast from extramammary primary lesion is a rare condition as its frequency fluctuates from 0.11% to 6.3% in histopathological series and 0.12% to 4.92% in radiological investigations; and the latter represent one of the largest case series available in the literature. Accordingly, the prevalent sources of metastasis are malignant melanoma of the skin and ovarian carcinoma, followed by atypical sources such as stomach, renal cell carcinoma, sarcoma, carcinoma of bronchus and lung, and carcinoma of larynx. Clinically, patients with metastases to the breast tend to display a solitary mass with rapid growth. In about half of the cases, tumors are adherent to the skin and superficially located; but pain, tenderness, nipple retraction, or discharge is not observed. Furthermore, the tumors are palpable in the upper outer quadrant and are bilateral in up to 25% of patients, whereas axillary node involvement is usually found in more than 50% of cases [1].

Here, we show examples of solitary lesions in six cases of ovarian cancer, tongue carcinoma, gastric carcinoma, and melanoma, whereas multiple lesions are demonstrated in four representative cases of ovarian cancer, gastric cancer, melanoma, and lymphoma. In accordance with the literature, more than half of the metastases described here were located superficially in subcutaneous tissue or immediately adjacent to the breast parenchyma, whereas enlarged axillary lymph nodes were observed in 8 cases, which were found to be more noticeable in patients with melanoma and ovarian cancer.

On the other hand, it is well documented that metastases to the breast can occur via two routes, the lymphatic and the hematogenous, and each metastasis shows different mammographic and sonographic appearance. For example, the well-circumscribed masses that have been associated with hematogenous dissemination are usually described as single or multiple, round to oval shaped tumors without desmoplastic reaction. Moreover, these masses are not associated with spiculations, architectural distortions, or microcalcifications. Contrary to this, the overall appearance of lymphatic metastases is hypoechoic masses associated with axillary or internal mammary lymph node enlargement, diffuse skin thickening, obliteration of subcutaneous fat, and lymphatic dilation secondary to mechanical obstruction of draining lymphatics [53]. It is suggested that the most common tumors that follow a spread pattern similar to the lymphatic metastases are the ovarian cancer and malignancies of the gastrointestinal tract [54]. Consistently, two of our cases of metastatic ovarian cancer exhibited the typical features of inflammatory carcinoma, supporting the notion that ovarian cancer cells preferentially metastasize via the lymphatic system due to the involvement of pelvic and para-aortic lymph nodes. However, more studies and observations are required to validate this hypothesis.

As regards the visualization of intramammary metastases by using contrast material-enhanced magnetic resonance imaging (MRI), it is proposed that the functional characteristics of the lesions, such as enhancement kinetics, may increase the specificity of the morphologic information regarding lesions. This observation is corroborated by the study of Surov et al. (2011), who were able to demonstrate that more than 80% of 93 intramammary metastases retrospectively viewed had type 2 and type 3 kinetic curves, which is indicative of malignancy [1].

By contrast, other reports indicate that intramammary metastases generally have slow or moderate initial enhancement rise [55], suggesting that these metastases can be easily misinterpreted as benign lesions. Therefore, the results available in the literature are not reproducible making a definitive conclusion premature.

In summary, due to the lack of specific radiographic signs, any newly developed tumor in a patient with a known history of extramammary malignancy should undergo biopsy for pathologic confirmation. Early and accurate diagnosis of secondary breast involvement is essential for appropriate management and for avoiding unnecessary and potentially harmful treatments in these patients.

## 4. Conclusion

Breast metastases that arise from extramammary malignancies are uncommon and usually related to a poor prognosis. The most common sources of breast metastases are lymphomas/leukemias, melanomas, and ovarian cancer. Due to the lack of specific radiographic signs, any newly developed masses in a patient with a known history of extramammary malignancy should undergo biopsy for pathologic confirmation. Establishing the actual rate of metastasis arising from breast cancer is difficult. However, there is evidence that in well-defined populations, such as those with ovarian cancer and melanoma, the incidence of breast metastases is soaring. Thus, medical experts are trying to find typical and atypical radiological features that suggest metastasis to the breast to guide diagnostic and therapeutic decisions.
